# Distinct metabolic phenotypes in adolescents with obesity identified by unsupervised learning: associations with insulin resistance and resting energy expenditure

**DOI:** 10.3389/fendo.2026.1875870

**Published:** 2026-08-27

**Authors:** Anelise Sonza, Sofia Tamini, Adele Bondesan, Diana Caroli, Laura Abbruzzese, Alessandro Sartorio

**Affiliations:** 1 Centro de Ciências da Saúde e do Esporte (CEFID), Department of Physical Therapy, Programa de Pós-Graduação em Ciências do Movimento Humano (PPGCMH), Universidade do Estado de Santa Catarina (UDESC), Florianópolis, Brazil; 2 Experimental Laboratory for Auxo-Endocrinological Research, Istituto Auxologico Italiano, Istituto di Ricovero e Cura a Carattere Scientifico (IRCCS), Piancavallo-Verbania, Italy; 3 Division of Auxology, Istituto Auxologico Italiano, Istituto di Ricovero e Cura a Carattere Scientifico (IRCCS), Piancavallo-Verbania, Italy

**Keywords:** clustering analysis, energy metabolism, insulin resistance, metabolic phenotyping, obesity, pediatric endocrinology

## Abstract

**Background:**

Pediatric obesity is characterized by substantial metabolic heterogeneity, including adiposity, insulin resistance, inflammation, and energy metabolism. Traditional categorical classifications may inadequately capture this complexity. Objective: To identify clinically meaningful metabolic phenotypes using unsupervised clustering and to compare the statistical performance and clinical interpretability of two- and three-cluster solutions.

**Methods:**

In this cross-sectional study, 758 children and adolescents with obesity underwent anthropometric, biochemical, and indirect calorimetry assessments. Fat-free mass was estimated using validated bioimpedance-based equations, and fat-free mass index (FFMI) was standardized before clustering. K-means clustering (k = 2 and k = 3) was performed using FFMI z-score, BMI-SDS, HOMA-IR, triglycerides, and HDL-cholesterol. Cluster validity was assessed using the Silhouette, Elbow, Davies–Bouldin index, Calinski–Harabasz index, and Gap statistic methods. Validation employed variables not included in clustering, such as glucose-insulin dynamics, inflammatory and hepatic markers, blood pressure, respiratory quotient, and resting energy expenditure (REE). Group comparisons were performed using Kruskal–Wallis and Dunn’s *post-hoc* tests with false discovery rate correction.

**Results:**

Both clustering solutions demonstrated significant separation across metabolic domains. The two-cluster model identified metabolically favourable and unfavourable phenotypes differing in adiposity, insulin resistance, inflammatory burden, hepatic markers, blood pressure, and REE. The three-cluster solution revealed a more granular metabolic stratification with an intermediate phenotype characterized by partial metabolic impairment. Validation confirmed robust differences across physiological variables (all FDR-adjusted p < 0.05). Although the two-cluster solution showed slightly superior internal validity, the three-cluster model provided greater clinical granularity and phenotypic resolution.

**Conclusions:**

Unsupervised clustering identified biologically coherent metabolic phenotypes in pediatric obesity. While the two-cluster solution provided statistically robust stratification, the three-cluster configuration captured a broader spectrum of metabolic heterogeneity and risk profiles, supporting the application of higher-resolution phenotyping approaches in pediatric endocrinology and metabolic risk assessment.

## 1 Introduction

Obesity during childhood and adolescence represents a major public health concern, with increasing prevalence worldwide and significant implications for long-term cardiometabolic health (1, 2). However, growing evidence suggests that obesity is not a homogeneous condition, particularly in pediatric populations (3), where individuals with similar degrees of adiposity may exhibit markedly different metabolic profiles (4).

Traditional metrics such as body mass index (BMI) fail to capture the complexity of body composition and metabolic regulation (5). In this context, fat-free mass (FFM) has emerged as a critical determinant of metabolic activity, given its strong association with resting energy expenditure (REE) and overall energy balance (6). The use of derived indices, such as the fat-free mass index (FFMI), allows normalization for body size and may provide additional insight into metabolic heterogeneity.

Moreover, insulin resistance (7), dyslipidemia (4, 8), low-grade inflammation, and hepatic dysfunction (9, 10) represent key components of obesity-related metabolic risk in adolescence. The coexistence of these factors varies substantially among individuals, giving rise to distinct phenotypes such as metabolically healthy obesity (MHO), metabolically unhealthy obesity (MUO) and intermediate patterns (4, 11, 12). Identifying these phenotypes during adolescence is particularly relevant, as this developmental stage is characterized by dynamic physiological changes that may influence long-term disease trajectories.

Unsupervised clustering approaches offer a data-driven strategy to identify such phenotypes by integrating multiple metabolic dimensions (11, 12). When combined with validation using other variables not included in the cluster and biomarkers, clustering can provide biologically meaningful classifications that extend beyond traditional definitions.

In addition, the relationship between metabolic phenotype and energy metabolism remains incompletely understood. REE, a major component of total energy expenditure, is closely linked to FFM but may also reflect underlying metabolic alterations, including insulin resistance and systemic inflammation.

Therefore, this study aimed to identify metabolic phenotypes in adolescents with obesity using body composition–derived indices and cardiometabolic markers, and to evaluate their association with REE and independent metabolic outcomes.

## 2 Methods

This is a cross-sectional analysis of retrospective data. This study was approved by the Ethics Committee No. 5, Lombardy Region, Italy (approval number 17/25; date of approval: March 25^th^, 2025; research code: 01C506; acronym: INDEMETSPED) and performed in compliance with the Declaration of Helsinki. All participants and their legal guardians had signed the written assent and the informed consent, respectively, at hospital admission, authorizing the anonymous use of all their clinical, anthropometric, and biochemical parameters for subsequent scientific purposes.

### 2.1 Study population

The study population included individuals with obesity (454 female and 303 male adolescents, aged 10–18 years), hospitalized at the Division of Auxology, Istituto Auxologico Italiano, IRCCS (Verbania–Piancavallo) for a 3-week, multidisciplinary body weight-reduction program.

Eligibility criteria included a BMI standard deviation score (SDS), calculated according to the Italian age- and sex-specific reference growth charts (BMI-SDS) ≥ 2.0 (13), primary (essential) obesity, and abstinence from alcohol consumption. Exclusion criteria comprised genetic or syndromic obesity, type 1 diabetes mellitus, hepatitis C virus infection, hepatitis B virus, and the presence of alcohol intake.

### 2.2 Metabolic and biochemical assessment

Fasting glucose, insulin, lipid profile (triglycerides, HDL, LDL), liver enzymes (ALT, AST, GGT), uric acid, and C-reactive protein (CRP) were measured using standard automated laboratory methods.

Insulin resistance was estimated using the homeostasis model assessment of insulin resistance (HOMA-IR), while β-cell function was estimated using HOMA-β.

An oral glucose tolerance test (OGTT) was performed, and glucose and insulin were measured at 0, 30, 60, 90, and 120 minutes.

Resting energy expenditure (REE) and respiratory quotient (RQ) were measured using indirect calorimetry. REE was expressed in kcal/day.

### 2.3 Anthropometry

Body weight and height were measured using standardized procedures. Body mass index (BMI) was calculated as weight (kg)/height (m²) and expressed as BMI standard deviation score (BMI-SDS) using age- and sex-adjusted reference values.

### 2.4 Body composition

Fat-free mass (FFM) was estimated using the prediction equation developed by Lazzer et al., which was cross-validated against dual-energy X-ray absorptiometry (DXA), a reference technique for measuring body composition, in pediatric populations with obesity (14).

The impedance index (ZI) was calculated as:


$$ZI=\frac{height^{2}(cm^{2})}{resistance\;\left(\Omega \right)}$$

where height was expressed in centimetres and resistance was measured at 50 kHz.

FFM was derived as:


$$FFM\;\left(kg\right)=0.87\times ZI+3$$

Fat-free mass index (FFMI) was calculated as:


$$FFMI=\frac{FFM}{height^{2}\left(m^{2}\right)}$$

FFMI was standardized using a z-score transformation across the entire study population (i.e., not sex-specific) before clustering, ensuring comparability of scale among clustering variables while preserving biological variability between males and females.

A sensitivity analysis was performed using the equation of Houtkooper et al. (15), commonly applied in pediatric populations.

### 2.5 Sexual maturity rating

Sexual maturity rating was assessed using Tanner staging by trained pediatric endocrinologists during clinical examination. Breast development in girls and genital development in boys, together with pubic hair development in both sexes, were classified into Tanner stages I–V according to the criteria originally described by Marshall and Tanner (16, 17).

### 2.6 Cluster analysis

Unsupervised machine learning was applied using k-means clustering to identify metabolic phenotypes.

Clustering was performed using k-means on five variables representing adiposity and metabolic status: FFMI z-score, BMI-SDS, HOMA-IR, triglycerides, and HDL-cholesterol. Before clustering, the complete feature matrix was standardized using a z-score transformation (mean = 0, standard deviation = 1) to ensure equal contribution of all five variables to Euclidean distance calculations.

The Hartigan–Wong algorithm was run with 50 random starts (nstart = 50) and a maximum of 10 iterations per initialization (iter.max = 10), consistent with the default implementation in R.

The optimal number of clusters was evaluated using the elbow method (within-cluster sum of squares), mean silhouette coefficient, Davies–Bouldin index, Calinski–Harabasz index and Gap statistic (500 bootstrap samples). All internal validity metrics consistently identified the two-cluster solution as the optimal statistical configuration.

Cluster stability was additionally evaluated using bootstrap resampling (500 iterations).

Cluster analyses were performed using the R packages *stats* (version 4.5.3; k-means algorithm), *cluster* (version 2.1.8.2; silhouette coefficient and Gap statistic), *clusterSim* (version 0.51-6; Davies–Bouldin and Calinski–Harabasz indices), *fpc* (version 2.2-14; bootstrap stability analysis), and *factoextra* (version 2.1.0; graphical visualization of cluster validity indices).

#### 2.6.1 Phenotypic validation using variables not included in cluster construction

To avoid circular inference, clusters were validated using variables not included in clustering: fasting glucose (FPG), OGTT glucose (120 min), fasting insulin (FPI), OGTT insulin (120 min), HOMA-β, C-reactive protein (CRP), uric acid, alanine aminotransferase (ALT), blood pressure (SBP, DBP), respiratory quotient (RQ), and measured REE (indirect calorimetry).

Differences between clusters were assessed using the Kruskal–Wallis test with a Benjamini–Hochberg false discovery rate (FDR) correction (5%).

Heatmaps summarizing the phenotypic profiles were generated using the R package *pheatmap* (version 1.0.13).

#### 2.6.2 Resting energy expenditure modelling

Resting energy expenditure (REE), measured by indirect calorimetry, was modelled using multivariable linear regression (ordinary least squares).

The primary predictor was FFM estimated using the Lazzer equation (14), previously validated against DXA in pediatric populations with obesity.

Three models were constructed:

• Model 1 (primary model):


REE ∼FFM+sex+age.

• Model 2 (body size–adjusted model):


REE ∼FFM+height+sex+age.

(to account for residual confounding by body size).

• Model 3 (sensitivity analysis):


REE ∼FFM (Houtkooper equation)+sex+age.

(to assess robustness to different FFM estimation methods).

Sex was coded as a binary variable (male = 1, female = 0).

Model assumptions were evaluated by inspecting the residual distribution, homoscedasticity (residual vs fitted plots), and multicollinearity (variance inflation factor).

To evaluate the robustness of the findings to the method used for estimating fat-free mass, a sensitivity analysis was performed using the bioimpedance equation proposed by Houtkooper et al., while the Lazzer equation remained the primary method because it was specifically developed and validated for pediatric populations with obesity. Model performance was assessed using adjusted R² and Akaike Information Criterion (AIC).

Linear regression analyses were performed using the base R *stats* package (version 4.5.3).

### 2.7 Statistical analysis

Given the non-normal distribution of most metabolic variables, group comparisons were performed using the Kruskal–Wallis rank sum test for global group differences, followed by Dunn’s *post-hoc* test for pairwise comparisons.

Multiple testing was controlled using the Benjamini–Hochberg false discovery rate (FDR 5%).

Continuous variables are presented as median (interquartile range). Cluster stability was assessed using multiple random initializations.

Given the known effects of puberty on insulin sensitivity, body composition, and energy metabolism, associations between cluster membership and pubertal maturation were further evaluated. For statistical analyses, Tanner stage was treated as an ordinal variable reflecting pubertal progression. Additional analyses were performed to evaluate the distribution of pubertal stages across clusters and to determine whether observed cluster differences were independent of pubertal maturation. Tanner stage distributions were compared using χ² tests, and age differences were assessed using Kruskal–Wallis tests. Multinomial logistic regression models were subsequently fitted to determine whether sex, Tanner stage, and age were independently associated with cluster membership. Additional analyses were performed to evaluate the distribution of pubertal stages across clusters and to determine whether observed cluster differences were independent of pubertal maturation.

Dunn’s multiple comparison tests were performed using the *FSA* package (version 0.10.1), whereas multinomial logistic regression analyses were performed using the *nnet* package (version 7.3-20). Data manipulation was performed using *dplyr* (version 1.2.1) and *tidyr* (version 1.3.2).

All analyses were conducted in R version 4.5.3 (R Foundation for Statistical Computing, Vienna, Austria).

## 3 Results

### 3.1 Resting energy expenditure modelling

Male sex was consistently associated with higher REE, whereas increasing age was associated with lower REE after adjustment for body composition (all p < 0.001). Adding height to the primary model resulted in a modest improvement in model fit. A sensitivity analysis using the Houtkooper equation produced results consistent with the primary analyses based on the Lazzer equation (**Supplementary Table 1**), supporting the robustness of the findings.

### 3.2 Internal validation of clustering solutions

All internal validity metrics consistently identified the two-cluster solution as the optimal statistical configuration. The elbow method (within-cluster sum of squares), mean silhouette coefficient (k=2: 0.252; k=3: 0.215), Davies–Bouldin index (k=2: 1.654; k=3: 1.673); Calinski-Harabasz index (k=2: 274.2; k=3: 216.7) and Gap statistic (500 bootstrap samples). Additional stability analyses using bootstrap resampling confirmed excellent reproducibility of the k=2 solution (Jaccard coefficients: 0.96 and 0.97). For the k=3 solution, the two extreme phenotypes remained stable (0.77 and 0.78), whereas the intermediate cluster showed lower stability (0.58). Despite lower internal validity, the three-cluster solution was retained for secondary analyses because it revealed an intermediate metabolic phenotype positioned between the two extreme groups across multiple metabolic domains. Therefore, k=3 should be interpreted as a clinically informative exploratory stratification rather than the statistically optimal clustering structure.

The two-cluster solution demonstrated excellent stability, with mean Jaccard coefficients of 0.96 and 0.97. For the three-cluster solution, two clusters showed good stability (0.77 and 0.78), whereas the intermediate cluster demonstrated lower stability (0.58).

### 3.3 Binary-cluster solution (k = 2)

The k=2 clustering solution identified two distinct metabolic phenotypes comprising 308 and 450 individuals (**Table 1**).

**Table 1:** Clinical and metabolic characteristics for binary-cluster (k = 2) solution.

Variable	Cluster 1 (n=308)	Cluster 2 (n=450)	p-value
SEX F	150 (48.7%)	304 (67.6%)	<0.001
SEX M	158 (51.3%)	146 (32.4%)	<0.001
FFMIz	0.72 (0.13-1.29)	-0.54 (-0.99--0.12)	<0.001
BMI-SDS	3.5 (3.2-3.7)	2.7 (2.4-3)	<0.001
HOMA-IR	3.7 (2.8-4.8)	2.1 (1.4-3)	<0.001
HOMA-β	369.2 (250.6-500.1)	224.2 (144.6-331.8)	<0.001
FPG	82 (78-86)	81 (77-85)	0.004
FPI	17.9 (14-23.6)	10.5 (7.1-15.1)	<0.001
CRP	0.4 (0.2-0.8)	0.3 (0.2-0.6)	<0.001
ALT	28 (20-42.5)	20 (15-29)	<0.001
SBP	130 (120-140)	120 (120-130)	<0.001
REE	2159 (1917-2397.5)	1769 (1587.5-1952.8)	<0.001

F, female; M, male; FFMIz, standardized fat-free mass index; BMI-SDS, standardized body mass index; HOMA-IR, Homeostasis Model Assessment - Insulin Resistance; HOMA-β, Homeostasis Model Assessment - β-cell function; FPG, fasting plasma glucose; FPI, fasting plasma insulin; CRP, C-reactive protein; ALT, alanine aminotransferase; SBP, systolic blood pressure; REE, resting energy expenditure.

Clusters differed significantly across all major metabolic domains. Cluster 1 exhibited a consistently more adverse metabolic profile compared with Cluster 2, including higher FFMI z-score, BMI-SDS, insulin resistance indices, inflammatory markers, liver enzymes, blood pressure, and REE.

Markers of insulin resistance showed particularly important separation. HOMA-IR, fasting insulin, and HOMA-β were markedly elevated in Cluster 1.

Inflammatory and hepatic markers (CRP and ALT) and systolic blood pressure were also significantly higher in Cluster 1.

Fasting plasma glucose differed statistically between clusters, but the clinical magnitude was limited.

Resting energy expenditure was significantly higher in Cluster 1. This finding is consistent with the higher fat-free mass and overall body size observed in this group.

A significant imbalance in sex distribution was observed (p < 0.001), with a higher proportion of females in Cluster 2. **Table 2** shows the two-cluster solution summary, where Cluster 1 is considered “Metabolically unfavourable” and Cluster 2 “Metabolically healthier”, considering cluster characteristics.

**Table 2:** Binary-Cluster´s summary.

Cluster 1 “metabolically unfavourable”	Cluster 2 “metabolically healthier”
• Higher FFMI • Higher BMI-SDS • Higher HOMA-IR • Higher triglycerides • Lower HDL • REE significantly higher (~2159 kcal)	• Lower adiposity • Lower insulin resistance • Better lipid profile • Lower REE (~1769 kcal)

FFMI, fat-free mass index; BMI-SDS, standardized body mass index; HOMA-IR, Homeostasis Model Assessment - Insulin Resistance; HDL, high-density lipoprotein; REE, resting energy expenditure.

**Figure 1:**
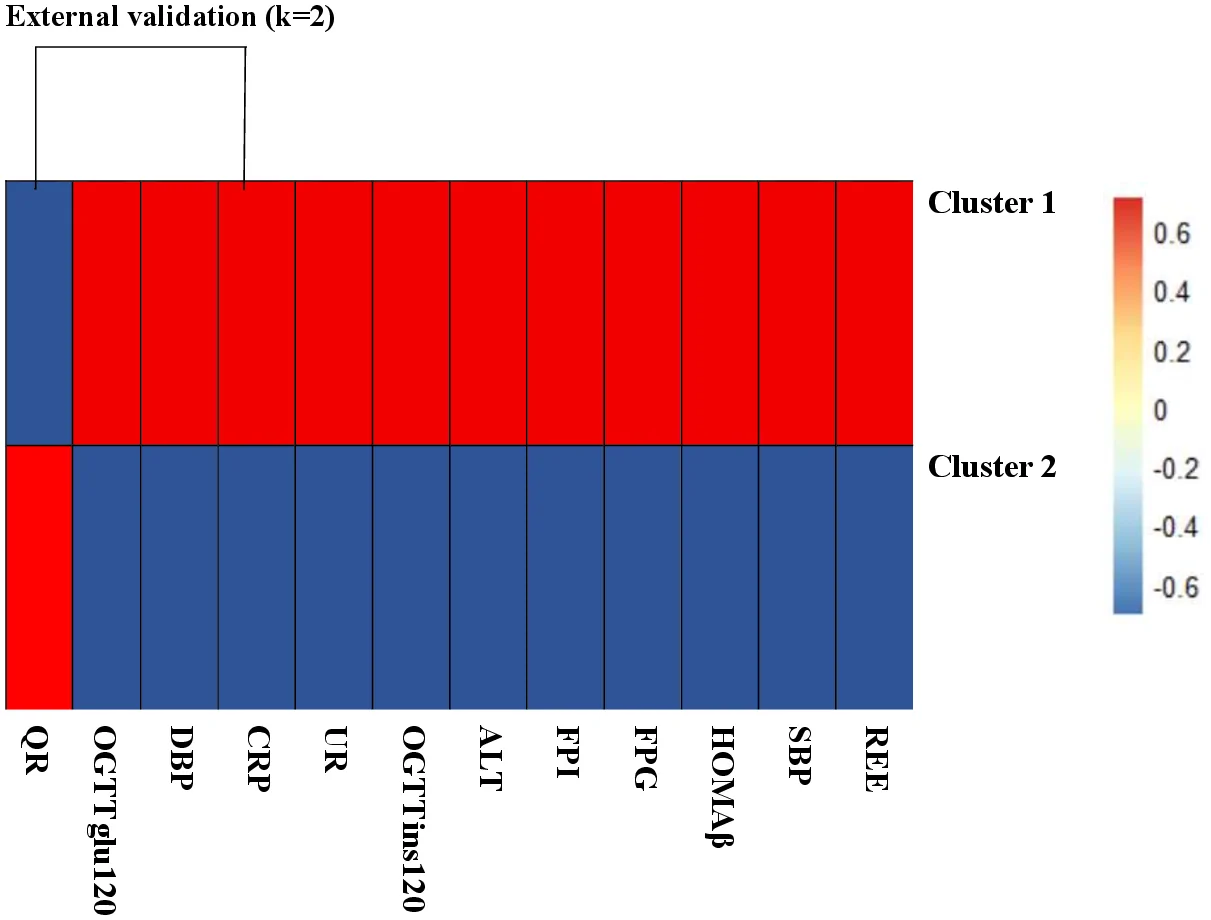
Validation heatmap with non-clustering variables (k = 2). The graded heatmap colours (red-white-blue) represent results ranging from “Metabolically unfavourable” (red) to “Metabolically favourable” (blue) across all measured variables. Participant clusters were identified exclusively using the k-means algorithm. The column dendrogram represents hierarchical clustering of the variables only and is included solely to facilitate visualization of similarity patterns among metabolic markers; it was not used to define or validate the k-means clusters. QR, respiratory quotient; OGTT, oral glucose tolerance test; DBP, diastolic blood pressure; CRP, C-reactive protein; UR, uric acid; FPI, fasting plasma insulin; FPG, fasting plasma glucose; HOMA-β, Homeostasis Model Assessment – β-cell function; SBP, systolic blood pressure; REE, resting energy expenditure.

Overall, the k = 2 solution delineates a clear dichotomy between a metabolically unfavourable, insulin-resistant phenotype and a comparatively healthier metabolic phenotype (**Figure 1**).

### 3.4 Three-cluster solution (k = 3)

**Figure 2:**
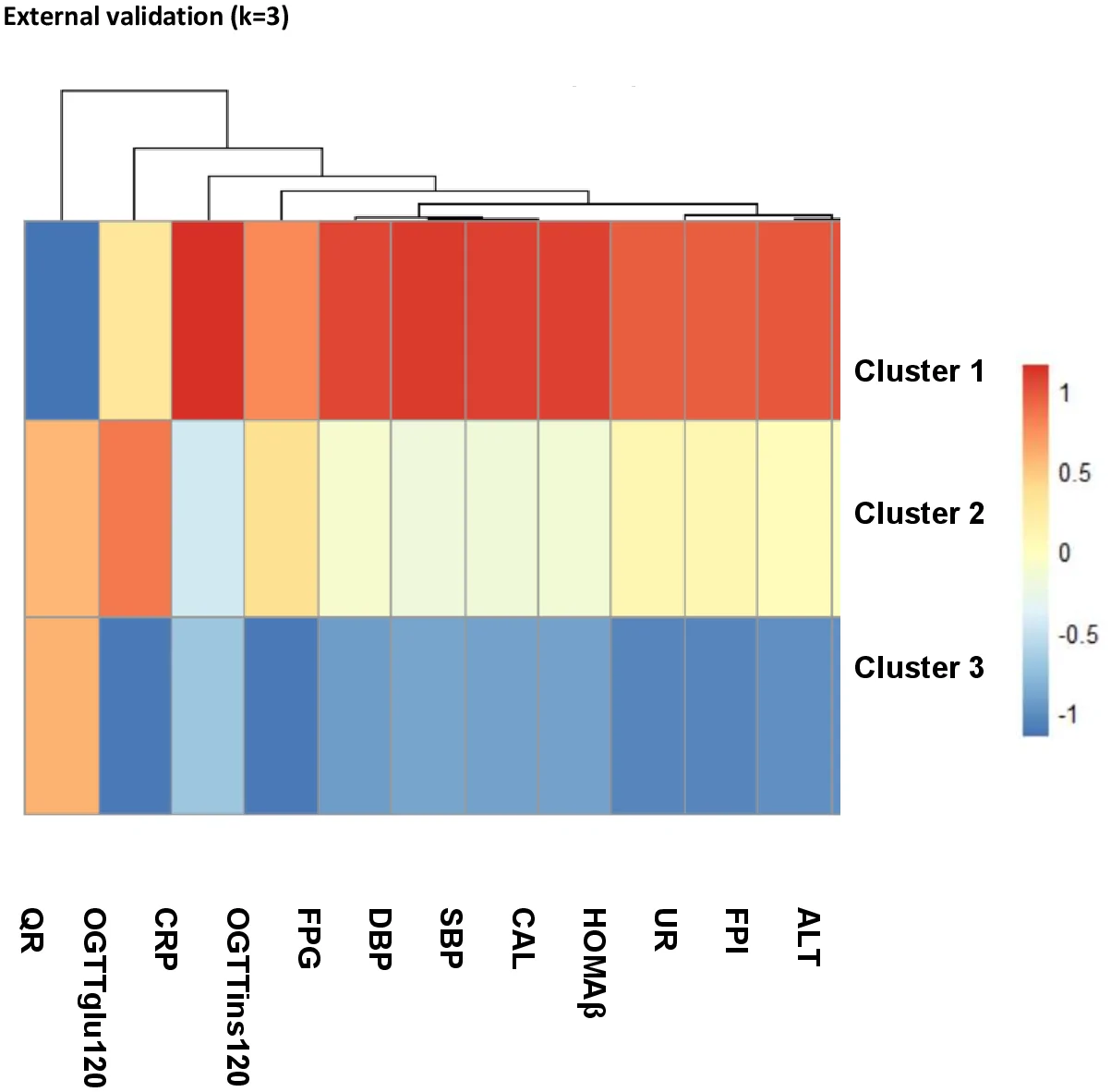
Validation heatmap with non-clustering variables (k = 3). The graded heatmap colours (red-white-blue) represent results ranging from “Metabolically unfavourable” (red), intermediate metabolic profile (white), to “Metabolically favourable” (blue) across all measured variables. Participant clusters were identified exclusively using the k-means algorithm. The column dendrogram represents hierarchical clustering of the variables only and is included solely to facilitate visualization of similarity patterns among metabolic markers; it was not used to define or validate the k-means clusters. QR, respiratory quotient; OGTT, oral glucose tolerance test; DBP, diastolic blood pressure; CRP, C-reactive protein; UR, uric acid; FPI, fasting plasma insulin; FPG, fasting plasma glucose; HOMA-β, Homeostasis Model Assessment – β-cell function; SBP, systolic blood pressure; REE, resting energy expenditure.

The k = 3 solution further stratified the cohort into three groups (n = 208, 180, and 370; **Table 3**), revealing a graded continuum of metabolic risk (**Figure 2**).

**Table 3:** Clinical and metabolic characteristics by three-cluster solution (k = 3).

Variable	Cluster 1 (n=208)	Cluster 2 (n=180)	Cluster 3 (n=370)	p-value
SEX F	97 (46.6%)	102 (56.7%)	255 (68.9%)	<0.001
SEX M	111 (53.4%)	78 (43.3%)	115 (31.1%)	<0.001
FFMIz	0.94 (0.49-1.58)	-0.28 (-0.72-0.27)	-0.53 (-1--0.09)	<0.001
BMI-SDS	3.58 (3.3-3.82)	3 (2.74-3.24)	2.7 (2.39-2.99)	<0.001
HOMA-IR	3.91 (2.95-5.22)	3.09 (2.18-3.97)	1.99 (1.32-2.72)	<0.001
HOMA-β	374.56(270.93-504.16)	328.25(222.49-440.8)	212.43(136.14-314.43)	<0.001
FPG	82 (78-86)	81.5 (77-86)	80.5 (77-85)	<0.001
OGTT 120 gly	116 (102-136)	120 (107-138)	112.5 (101-126)	<0.001
FPI	19.2 (14.9-25.38)	15.25 (11.17-19.8)	9.8 (6.8-14)	<0.001
OGTT 120 ins	81.8 (57.65-142.25)	85.65 (58.08-119.25)	62.25 (41.92-88.77)	<0.001
CRP	0.4 (0.3-0.8)	0.3 (0.2-0.7)	0.3 (0.2-0.6)	<0.001
ALT	28 (21-44.25)	23 (16.75-38)	19 (15-28)	<0.001
SBP	130 (120-140)	120 (120-130)	120 (120-130)	<0.001
REE	2225 (2010.75-2453.75)	1902.5 (1760.75-2124.5)	1754.5 (1561.25-1949.75)	<0.001

F, female; M, male; FFMIz, standardized fat-free mass index; BMI-SDS, standardized body mass index; HOMA-IR, Homeostasis Model Assessment - Insulin Resistance; HOMA-β, Homeostasis Model Assessment β-cell function; FPG, fasting plasma glucose; FPI, fasting plasma insulin; OGTT, oral glucose tolerance test; CRP, C-reactive protein; ALT, alanine aminotransferase; SBP, systolic blood pressure; REE, resting energy expenditure.

A consistent stepwise pattern was observed across nearly all variables.

Considering adiposity and body composition, FFMI z-score and BMI-SDS decreased progressively from Cluster 1 to Cluster 3.

For insulin resistance and β-cell function, a clear gradient was evident, highest in Cluster 1, intermediate in Cluster 2 and lowest in Cluster 3. This pattern was consistent across HOMA-IR, fasting insulin, OGTT-derived insulin responses, and HOMA-β.

The glycemic profile, fasting glucose, and OGTT 120-min glucose showed statistically significant differences across clusters, although absolute differences remained modest.

Inflammation and liver function (CRP and ALT) decreased progressively across clusters. Also, for cardiovascular parameters, systolic blood pressure showed a similar downward trend, supporting the cardiometabolic coherence of the clustering structure.

For energy metabolism, REE showed a marked stepwise decrease from Cluster 1 to Cluster 3.A significant gradient in sex distribution was observed (p < 0.001), with an increasing proportion of females from Cluster 1 to Cluster 3. **Table 4** shows the three-cluster solution summary, where Cluster 1 is considered “Severe insulin-resistant hypermetabolic phenotype”, Cluster 2 the “Intermediate cardiometabolic risk phenotype”, and Cluster 3 the “Metabolically healthier insulin-sensitive phenotype”, considering cluster characteristics.

**Table 4:** Three-cluster solution summary.

Cluster 1 - “severe insulin-resistant hypermetabolic phenotype”	Cluster 2 - “intermediate cardiometabolic risk phenotype”	Cluster 3 - “metabolically healthier insulin-sensitive phenotype”
• Highest FFMI and BMI-SDS (marked adiposity with increased lean mass component) • Highest HOMA-IR and fasting insulin levels (most severe insulin resistance profile) • Highest β-cell activity (HOMA-β markedly elevated, compensatory hyperinsulinemia) • Higher inflammatory burden (elevated CRP compared to other clusters) • Higher liver enzyme levels (ALT, suggesting greater metabolic liver stress) • Higher OGTT-derived insulin and glucose responses (greater postprandial metabolic dysregulation) • Highest REE(~2225 kcal), indicating a hypermetabolic state	• Moderately elevated BMI-SDS and FFMI (intermediate adiposity profile) • Intermediate insulin resistance (HOMA-IR between Cluster 1 and 3) • Partial β-cell compensation (HOMA-β reduced compared to Cluster 1) • Intermediate lipid and inflammatory profile (CRP and ALT moderately elevated) • Intermediate REE (~1902 kcal) • Mixed glycemic response during OGTT, suggesting transitional metabolic state	• Lowest BMI-SDS and FFMI (leaner phenotype with lower adiposity burden) • Lowest HOMA-IR and fasting insulin (most insulin-sensitive group) • Lowest β-cell demand (reduced compensatory insulin secretion) • Lower inflammatory markers (lowest CRP among clusters) • Lower liver enzyme levels (more favourable hepatic metabolic profile) • Most favourable OGTT glucose and insulin responses • Lowest REE (~1754 kcal), consistent with a metabolically efficient state

FFMI, fat-free mass index; BMI-SDS, standardized body mass index; HOMA-IR, Homeostasis Model Assessment - Insulin Resistance; HOMA-β, Homeostasis Model Assessment β-cell function; FPG, fasting plasma glucose; FPI, fasting plasma insulin; OGTT, oral glucose tolerance test; CRP, C-reactive protein; ALT, alanine aminotransferase; REE, resting energy expenditure.

Kruskal–Wallis analyses demonstrated significant differences across all externally validated variables after Benjamini-Hochberg correction (adjusted p ≤ 0.042). Test statistics ranged from χ² = 6.32 for fasting plasma glucose to χ² = 345.28 for BMI-SDS. Detailed results are provided in **Supplementary Table 2**. The complete Dunn *post-hoc* comparisons with Benjamini–Hochberg-adjusted p-values are reported in **Supplementary Table 3**.

### 3.5 Phenotypic validation using variables not included in cluster construction

Validation using variables not included in clustering confirmed the separation between clusters.

In the k = 2 solution, clusters differed significantly in fasting glucose, OGTT glucose (120 min), fasting insulin and OGTT insulin (120 min), HOMA-β, CRP, uric acid, ALT, blood pressure, respiratory quotient, and REE (all FDR-adjusted p < 0.05).

Similarly, in the k = 3 solution, significant differences were observed among variables not used for clustering, with a consistent stepwise pattern across clusters. In particular, insulin-related parameters (fasting insulin, OGTT insulin, and HOMA-β), inflammatory markers (CRP), hepatic enzymes (ALT), and cardiovascular measures (blood pressure) showed a graded distribution, mirroring the continuum of metabolic risk identified by the clustering model.

The k = 3 solution provided additional resolution by capturing intermediate metabolic alterations that were not distinguishable in the binary model.

### 3.6 Comparison between k = 2 and k = 3 solutions

Both clustering solutions showed statistically significant differences across anthropometric, metabolic, inflammatory, hepatic, cardiovascular, and energy metabolism variables (**Tables 1**, **3**).

The k = 2 solution separated participants into two metabolically distinct groups, whereas the k = 3 solution identified an additional intermediate cluster. This intermediate cluster displayed values for insulin resistance (HOMA-IR, fasting insulin), β-cell function (HOMA-β), inflammatory markers (CRP), hepatic marker (ALT), and resting energy expenditure that were consistently positioned between the two extreme clusters.

Internal validation indices favored the k = 2 solution (silhouette coefficient: 0.252 vs. 0.215 (**Supplementary Figure 1**); Davies–Bouldin index: 1.654 vs. 1.673; Calinski–Harabasz index: 274.2 vs. 216.7. Also, Gap statistic: optimal at k = 2 and Elbow method (**Supplementary Figure 1**), whereas the k = 3 solution provided additional phenotypic stratification through identification of an intermediate metabolic group, that may be clinically informative. The relatively modest silhouette coefficients observed in both models suggest partial overlap between metabolic phenotypes and support the interpretation of metabolic risk as a continuum rather than as sharply separated categories. Nevertheless, the lower silhouette coefficient indicates reduced internal cohesion, suggesting a trade-off between statistical robustness (k = 2) and clinical interpretability (k = 3).

Because adolescence is characterized by major physiological changes in body composition and insulin sensitivity, the relationship between cluster membership and pubertal maturation was specifically examined. Tanner stage distribution differed significantly between the two clustering solutions (k=2: χ²=34.0, p<0.001; k=3: χ²=25.8, p=0.001), and median age also differed among clusters (all p<0.001).

To determine whether cluster allocation primarily reflected pubertal development, multinomial logistic regression models were fitted that included sex, Tanner stage, and age. In the k=2 solution, male sex was independently associated with the metabolically unfavourable cluster (OR = 2.71, p<0.001), whereas Tanner stage showed a modest association (OR = 1.32, p=0.011) and age was not significant (p=0.175). Similar findings were observed in the k=3 solution. Male sex remained strongly associated with Cluster 1 versus Cluster 3 (OR = 3.15, p<0.001) and Cluster 2 versus Cluster 3 (OR = 1.84, p=0.002). Tanner stage showed only a modest association with Cluster 1 (OR = 1.36, p=0.019) and no significant association with Cluster 2 (p=0.368), whereas age was not independently associated with cluster membership. These findings indicate that the identified metabolic phenotypes cannot be explained solely by pubertal maturation.

## 4 Discussion

This study demonstrates that unsupervised clustering applied to a multidimensional endocrine–metabolic dataset can identify distinct and biologically coherent metabolic phenotypes in children and adolescents with obesity. For pediatric populations, unsupervised machine clustering has been successfully used to identify subtypes in Metabolically Dysfunctional-Associated Steatotic Liver Disease (18), but also in other populations and diseases (19, 20).

### 4.1 k = 2 and k = 3 clustering solutions

Across both clustering solutions, consistent intergroup differences were observed in insulin resistance, body composition, inflammatory markers, hepatic function, cardiovascular parameters, and energy metabolism, supporting the presence of a structured metabolic heterogeneity rather than random variation. A review (21) shows that pediatric obesity is a complex disease involving multiple organs and systems, with metabolic and inflammatory complications, supporting a multidimensional approach.

The binary clustering solution (k = 2) identified two clearly distinct phenotypes broadly corresponding to metabolically unfavourable and relatively healthier profiles. This dichotomous structure aligns with conventional clinical frameworks that stratify individuals into high- and low-risk categories and was supported by slightly better internal validity metrics. However, such a binary classification may inadequately capture the dynamic and continuous nature of metabolic regulation during growth and pubertal development (22). In pediatric populations, endocrine and metabolic processes evolve along trajectories influenced by maturation, body composition changes, and hormonal modulation, which are not inherently dichotomous (23). An important consideration is that internal validation metrics indicated only moderate cluster separation. Mean silhouette coefficients were 0.25 for the k=2 solution and 0.21 for the k=3 solution. Considering the cluster stability through Jaccard coefficients, the findings support the two-cluster solution as the statistically most robust configuration while indicating that the intermediate cluster identified in the k=3 solution may represent a transitional metabolic state rather than a fully discrete phenotype. Also, both below commonly cited thresholds for well-separated clusters. However, this finding is biologically plausible in the context of pediatric obesity, where insulin resistance, adiposity, inflammation, and energy expenditure are continuously distributed traits rather than discrete entities. Therefore, the observed overlap between clusters likely reflects the continuum of metabolic risk present within the study population. Consequently, the identified clusters should not be interpreted as strictly distinct biological categories, but rather as data-driven phenotypic groupings that represent different positions along a metabolic severity spectrum.

Both clustering solutions revealed highly significant intergroup differences across metabolic, hormonal, inflammatory, and cardiovascular domains (all global p < 0.01, most p < 0.001), confirming robust cluster separation. In this context, the three-cluster solution provides a more nuanced representation of metabolic heterogeneity. The identification of an intermediate phenotype, characterized by moderate insulin resistance, partial β-cell compensation, and intermediate inflammatory and hepatic profiles, supports the concept of a metabolic continuum rather than discrete categories. This finding is particularly relevant in pediatric endocrinology, where transitional states often precede overt metabolic dysfunction and may represent critical windows for early intervention (24). The graded distribution observed across clusters for insulin-related variables, inflammatory markers, and hepatic enzymes further reinforces the biological plausibility of this continuum model. The present findings can also be interpreted within the framework of metabolically healthy obesity (MHO) and metabolically unhealthy obesity (MUO), concepts that have been widely used to describe heterogeneity in pediatric obesity. The k=2 solution broadly mirrors this paradigm, separating participants into a metabolically less adverse phenotype and a metabolically unfavourable phenotype characterized by higher insulin resistance, inflammation, hepatic biomarkers, blood pressure, and energy expenditure. However, the k=3 solution revealed an intermediate phenotype that cannot be readily classified as either MHO or MUO. Interestingly, the intermediate phenotype identified in the three-cluster solution demonstrated lower bootstrap stability than the two extreme clusters. Rather than indicating a methodological weakness, this finding may reflect the biological reality of metabolic progression, in which individuals transition gradually between metabolically favourable and unfavourable states. Thus, the reduced stability of the intermediate cluster may be consistent with a continuum model of metabolic risk rather than the existence of sharply separated categories. This observation supports the notion that metabolic risk in pediatric obesity is better conceptualized as a continuum rather than a dichotomous state and suggests that conventional MHO/MUO definitions may oversimplify the underlying biological complexity.

### 4.2 Insulin resistance–related variables

One of the principal findings of this study is that the identified metabolic phenotypes were primarily distinguished by a coordinated gradient in insulin resistance. Although cluster formation incorporated five equally standardized variables, the most consistent differences observed across the resulting phenotypes involved insulin resistance-related measures, including HOMA-IR, fasting insulin, HOMA-β, and OGTT-derived insulin responses. Furthermore, independent validation variables not used during clustering—including glucose tolerance, inflammatory markers, hepatic enzymes, blood pressure, respiratory quotient, and resting energy expenditure—followed the same progressive pattern across clusters. Together, these findings suggest that insulin resistance represents the central physiological axis underlying the metabolic heterogeneity observed in this cohort, rather than an isolated metabolic abnormality. This observation is consistent with the known role of insulin as a pleiotropic hormone that regulates not only glucose metabolism but also protein synthesis, adipose tissue function, and growth-related energy partitioning (25). The parallel gradients observed in the FFMI z-score and BMI-SDS suggest that alterations in insulin sensitivity are closely linked to differences in both fat mass and lean mass accrual during growth.

### 4.3 Pubertal maturation

Pubertal maturation represents a potential source of confounding in pediatric metabolic studies because insulin sensitivity, body composition, and energy expenditure undergo substantial physiological changes throughout adolescence. Consistent with this, Tanner stage distribution differed across clusters in both clustering solutions. However, after simultaneous adjustment for sex, Tanner stage, and age, the association between cluster membership and pubertal status was markedly attenuated. Tanner stage showed only modest associations with the most metabolically unfavourable phenotypes, whereas chronological age was not independently associated with cluster allocation. In contrast, sex remained consistently associated with cluster membership across both clustering solutions. These findings suggest that the identified phenotypes capture metabolic heterogeneity that extends beyond normal maturational variation and support the interpretation that the clustering structure reflects genuine biological differences in metabolic regulation rather than merely differences in pubertal development. Although BMI-SDS and FFMI are correlated measures of body size, they reflect distinct physiological dimensions of obesity. BMI-SDS primarily captures relative adiposity, whereas FFMI reflects variation in lean tissue mass, which is a major determinant of energy expenditure and metabolic demand. Both variables were therefore retained to provide a more comprehensive representation of body composition.

### 4.4 Resting energy expenditure

In all three multivariable linear regression models, estimated fat-free mass remained significantly associated with measured resting energy expenditure after adjustment for sex, age, and, where applicable, height (all p < 0.001).

Resting energy expenditure (REE) also differed significantly across clusters, showing a progressive increase with worsening metabolic profile. Specifically, REE showed a progressive decrease from Cluster 1 to Cluster 3, indicating that the identified phenotypes differed not only in metabolic risk but also in overall energy metabolism. However, this finding should be interpreted cautiously, as REE is strongly determined by fat-free mass, which represents the principal determinant of energy expenditure at rest (6, 26, 27).

Nevertheless, the parallel gradients observed for REE, insulin resistance, adiposity, and inflammatory markers indicate that energy metabolism is integrated within the broader metabolic phenotype identified by the clustering analysis.

### 4.5 Inflammatory and hepatic markers

The observed differences in inflammatory and hepatic markers further support the biological validity of the clustering structure. The progressive increase in CRP and ALT across clusters is consistent with the presence of low-grade systemic inflammation and hepatic metabolic stress, both of which are recognized components of early metabolic dysfunction in pediatric obesity (28, 29). Similarly, the stepwise differences in systolic blood pressure indicate that cardiovascular alterations are already detectable along this metabolic spectrum, reinforcing the concept that these clusters capture a multidimensional cardiometabolic phenotype rather than isolated abnormalities.

### 4.6 Sex distribution across clusters

An important consideration is the observed imbalance in sex distribution across clusters, with a higher proportion of females in the metabolically healthier groups. Sex-related physiological differences in adolescents have already been published (7, 30, 31). This finding also suggests that sexual dimorphism, including body composition, pubertal timing, and hormonal milieu, may influence cluster allocation. Although sex was accounted for in regression models, residual confounding cannot be excluded, and this aspect should be further explored in future studies.

From a methodological perspective, this study highlights the distinction between statistical optimality and clinical interpretability. While the k = 2 solution demonstrated slightly better internal validity, the k = 3 solution provided a more nuanced and clinically informative representation of metabolic heterogeneity. This trade-off is particularly relevant in biomedical applications, where the goal is not only to optimize mathematical criteria but also to generate clinically meaningful classifications. Our findings are also consistent with emerging evidence supporting clustering-based approaches in pediatric obesity research. Recently, Kahraman et al. (32) applied multivariate cluster analysis to children with obesity and demonstrated that systolic and diastolic blood pressure clustered with different anthropometric, nutritional, and behavioural determinants, highlighting the multifactorial nature of obesity-related cardiovascular risk. Their study showed that cluster analysis can identify clinically meaningful patterns that are not readily apparent using conventional univariate approaches. Similarly, the present study revealed coherent clustering among insulin resistance, adiposity, inflammation, hepatic function, and energy metabolism, supporting the value of data-driven phenotyping for uncovering latent biological heterogeneity within pediatric obesity.

### 4.7 Limitations

Several limitations should be acknowledged. The cross-sectional design precludes inference regarding temporal progression between metabolic phenotypes, and longitudinal studies are needed to determine whether individuals transition between clusters over time. In addition, the use of bioimpedance-derived equations to estimate fat-free mass, although validated in pediatric populations, may introduce measurement error compared with gold-standard techniques such as DXA. However, sensitivity analyses using alternative equations yielded consistent findings, supporting the robustness of the results. Validation was performed using different metabolic variables within the same cohort, but replication in external populations is necessary to establish generalizability. Finally, while the k = 3 solution enhances clinical interpretability, it also introduces greater complexity, which may limit immediate applicability in routine clinical settings.

## 5 Conclusion

This study demonstrates that unsupervised clustering of multidimensional endocrine-metabolic data identifies clinically meaningful phenotypes within pediatric obesity. Although the two-cluster solution provided the strongest internal validity, an exploratory three-cluster solution provided additional phenotypic granularity by identifying an intermediate metabolic profile positioned between the two extremes of metabolic risk. The identified phenotypes were consistently differentiated across multiple metabolic domains, including insulin dynamics, inflammation, hepatic biomarkers, cardiovascular parameters, and energy metabolism.

The relatively modest cluster validity indices suggest that metabolic phenotypes in pediatric obesity are characterized by partial overlap rather than discrete boundaries, supporting the concept of a continuum of metabolic risk. Within this framework, the three-cluster solution should be interpreted as a clinically informative stratification of a continuous biological process rather than as evidence of completely distinct metabolic subgroups.

Overall, these findings highlight the value of data-driven phenotyping approaches for improving the characterization of metabolic heterogeneity in pediatric obesity. Future longitudinal studies are needed to determine the temporal stability of these phenotypes, their relationship with clinical outcomes, and their potential role in personalized prevention and treatment strategies in pediatric endocrinology.

## Data Availability

The datasets presented in this study can be found in online repositories. Raw data will be available upon a reasonable request to the corresponding author (ASo) and ASa.
