# Supplementary material for: Distinct metabolic phenotypes in adolescents with obesity identified by unsupervised learning: associations with insulin resistance and resting energy expenditure
Source: Front Endocrinol (Lausanne). 2026 Aug 27;17:1875870. doi: 10.3389/fendo.2026.1875870 (PMC13557994; doi:10.3389/fendo.2026.1875870)
Supplement: Supplementary file 1 [file Table1.docx]

**Supplementary Table S1. Sensitivity analyses of resting energy expenditure models using alternative fat-free mass prediction equations.**

|  | **Model 1** | **Model 2** | **Model 3** |
| --- | --- | --- | --- |
| **Predictor** | Primary analysis (Lazzer equation) | Adjustment for body size | Sensitivity analysis (Houtkooper equation) |
| **Intercept** | 754.65 (623.21 to 886.08), p < 0.001 | 54.30 (−319.64 to 428.23), p = 0.776 | 787.48 (670.08 to 904.87), p < 0.001 |
| **Fat-free mass** | 26.64 (24.37 to 28.91), p < 0.001 | 22.58 (19.54 to 25.61), p < 0.001 | 24.53 (22.88 to 26.17),  p < 0.001* |
| **Height (m)** | — | 607.08 (303.22 to 910.93), p < 0.001 | — |
| **Male sex** | 142.19 (102.38 to 182.00), p < 0.001 | 132.32 (92.58 to 172.07), p < 0.001 | 114.93 (79.44 to 150.41), p < 0.001 |
| **Age (years)** | −18.79 (−28.39 to −9.20), p < 0.001 | −24.35 (−34.25 to −14.44), p < 0.001 | −29.94 (−38.62 to −21.26), p < 0.001 |
| **Adjusted R²** | 0.579 | 0.587 | 0.664 |
| **AIC** | 10466.65 | 10453.32 | 10295.94 |
| **BIC** | 10489.80 | 10481.11 | 10319.09 |

*Fat-free mass estimated using the Houtkooper equation.

Legend: Values are regression coefficients (β) with 95% confidence intervals. Model 1 included fat-free mass estimated using the Lazzer equation, sex, and age. Model 2 additionally adjusted for height. Model 3 was a sensitivity analysis replacing the Lazzer equation with the Houtkooper equation for fat-free mass estimation. Model performance was evaluated using adjusted R², Akaike Information Criterion (AIC), and Bayesian Information Criterion (BIC). Lower AIC/BIC and higher adjusted R² indicate better model fit.
