# Supplementary material for: Distinct metabolic phenotypes in adolescents with obesity identified by unsupervised learning: associations with insulin resistance and resting energy expenditure
Source: Front Endocrinol (Lausanne). 2026 Aug 27;17:1875870. doi: 10.3389/fendo.2026.1875870 (PMC13557994; doi:10.3389/fendo.2026.1875870)
Supplement: Supplementary file 2 [file Table2.docx]

**Supplementary Table S2. Global Kruskal–Wallis comparisons across the three-cluster solution (k = 3).**

| **Variable** | **χ²** | **df** | **Raw p-value** | **FDR-adjusted p-value** |
| --- | --- | --- | --- | --- |
| FFMI z-score | 330.10 | 2 | 2.09×10⁻⁷² | 1.19×10⁻⁷¹ |
| BMI-SDS | 345.28 | 2 | 1.06×10⁻⁷⁵ | 1.80×10⁻⁷⁴ |
| HOMA-IR | 196.84 | 2 | 1.81×10⁻⁴³ | 5.12×10⁻⁴³ |
| Triglycerides | 330.27 | 2 | 1.92×10⁻⁷² | 1.19×10⁻⁷¹ |
| HDL-cholesterol | 201.08 | 2 | 2.17×10⁻⁴⁴ | 9.23×10⁻⁴⁴ |
| Fasting plasma glucose | 6.32 | 2 | 0.042 | 0.042 |
| OGTT glucose (120 min) | 19.15 | 2 | 6.93×10⁻⁵ | 8.41×10⁻⁵ |
| Fasting insulin | 199.64 | 2 | 4.45×10⁻⁴⁴ | 1.51×10⁻⁴³ |
| OGTT insulin (120 min) | 59.06 | 2 | 1.50×10⁻¹³ | 2.12×10⁻¹³ |
| HOMA-β | 131.42 | 2 | 2.89×10⁻²⁹ | 5.47×10⁻²⁹ |
| CRP | 17.55 | 2 | 1.54×10⁻⁴ | 1.75×10⁻⁴ |
| Uric acid | 136.24 | 2 | 2.60×10⁻³⁰ | 5.53×10⁻³⁰ |
| ALT | 64.60 | 2 | 9.40×10⁻¹⁵ | 1.45×10⁻¹⁴ |
| Systolic blood pressure | 82.17 | 2 | 1.43×10⁻¹⁸ | 2.44×10⁻¹⁸ |
| Diastolic blood pressure | 43.71 | 2 | 3.22×10⁻¹⁰ | 4.22×10⁻¹⁰ |
| Respiratory quotient | 11.74 | 2 | 0.0028 | 0.0030 |
| Resting energy expenditure | 195.39 | 2 | 3.74×10⁻⁴³ | 9.07×10⁻⁴³ |

Abbreviations: FFMI, fat-free mass index; BMI-SDS, body mass index standard deviation score; HOMA-IR, Homeostasis Model Assessment of Insulin Resistance; HDL, High Density Lipoprotein; HOMA-β, Homeostasis Model Assessment of β-cell function; CRP, C-reactive protein; ALT, alanine aminotransferase; OGTT, oral glucose tolerance test.
