# Supplementary material for: Distinct metabolic phenotypes in adolescents with obesity identified by unsupervised learning: associations with insulin resistance and resting energy expenditure
Source: Front Endocrinol (Lausanne). 2026 Aug 27;17:1875870. doi: 10.3389/fendo.2026.1875870 (PMC13557994; doi:10.3389/fendo.2026.1875870)
Supplement: Supplementary file 3 [file Table3.docx]

**Supplementary Table S3. Dunn post-hoc pairwise comparisons with Benjamini-Hochberg correction for the three-cluster solution (k = 3)**

| **Variable** | **C1 vs C2** | **C1 vs C3** | **C2 vs C3** |
| --- | --- | --- | --- |
| FFMI z-score | Significant | Significant | Significant |
| BMI-SDS | Significant | Significant | Significant |
| HOMA-IR | Significant | Significant | Significant |
| TGL | Significant | Significant | Significant |
| HDL-cholesterol | Significant | Significant | Significant |
| Fasting plasma glucose | NS | Significant | NS |
| OGTT glucose (120 min) | NS | Significant | Significant |
| Fasting insulin | Significant | Significant | Significant |
| OGTT insulin (120 min) | NS | Significant | Significant |
| HOMA-β | NS | Significant | Significant |
| CRP | Significant | Significant | NS |
| UR | Significant | Significant | Significant |
| ALT | Significant | Significant | Significant |
| SBP | Significant | Significant | Significant |
| DBP | Significant | Significant | NS |
| RQ | Significant | Significant | NS |
| REE | Significant | Significant | Significant |

NS = not significant after Benjamini–Hochberg correction.

| **Variable** | **Comparison** | **Z** | **Adjusted p** |
| --- | --- | --- | --- |
| Variable | Comparison | Z | Adjusted p |
| FFMI_z | C1 vs C2 | 11.769386 | 8.419772e-32 |
| FFMI_z | C1 vs C3 | 18.019107 | 4.138597e-72 |
| FFMI_z | C2 vs C3 | 3.999535 | 6.346715e-05 |
| BMI.SDS | C1 vs C2 | 10.547899 | 7.791858e-26 |
| BMI.SDS | C1 vs C3 | 18.574744 | 1.543509e-76 |
| BMI.SDS | C2 vs C3 | 5.897751 | 3.684902e-09 |
| HOMA.IR | C1 vs C2 | 4.390251 | 1.132199e-05 |
| HOMA.IR | C1 vs C3 | 13.565302 | 1.929439e-41 |
| HOMA.IR | C2 vs C3 | 8.018443 | 1.606406e-15 |
| TGL | C1 vs C2 | -10.207922 | 2.741056e-24 |
| TGL | C1 vs C3 | 7.043936 | 1.868846e-12 |
| TGL | C2 vs C3 | 18.152549 | 3.677366e-73 |
| HDL | C1 vs C2 | 6.249909 | 4.106917e-10 |
| HDL | C1 vs C3 | -7.266540 | 5.532199e-13 |
| HDL | C2 vs C3 | -13.930985 | 1.231953e-43 |
| FPG | C1 vs C2 | 0.913373 | 3.610462e-01 |
| FPG | C1 vs C3 | 2.461645 | 4.149050e-02 |
| FPG | C2 vs C3 | 1.324357 | 2.780766e-01 |
| OGTT.glic120 | C1 vs C2 | -1.757764 | 7.878769e-02 |
| OGTT.glic120 | C1 vs C3 | 2.402742 | 2.440899e-02 |
| OGTT.glic120 | C2 vs C3 | 4.260449 | 6.120491e-05 |
| FPI | C1 vs C2 | 4.322937 | 1.539660e-05 |
| FPI | C1 vs C3 | 13.634729 | 7.467385e-42 |
| FPI | C2 vs C3 | 8.160060 | 5.022885e-16 |
| OGTT.ins120 | C1 vs C2 | 0.288727 | 7.727907e-01 |
| OGTT.ins120 | C1 vs C3 | 6.596406 | 1.263735e-10 |
| OGTT.ins120 | C2 vs C3 | 5.967198 | 3.620435e-09 |
| HOMA.Beta | C1 vs C2 | 2.936407 | 3.320388e-03 |
| HOMA.Beta | C1 vs C3 | 10.891757 | 3.785445e-27 |
| HOMA.Beta | C2 vs C3 | 7.097456 | 1.906117e-12 |
| CRP | C1 vs C2 | 2.665699 | 1.152427e-02 |
| CRP | C1 vs C3 | 4.162800 | 9.431066e-05 |
| CRP | C2 vs C3 | 0.983665 | 3.252801e-01 |
| UR | C1 vs C2 | 3.544788 | 3.929290e-04 |
| UR | C1 vs C3 | 11.256348 | 6.465183e-29 |
| UR | C2 vs C3 | 6.763624 | 2.018727e-11 |
| ALT | C1 vs C2 | 3.333301 | 8.582199e-04 |
| ALT | C1 vs C3 | 7.948468 | 5.664966e-15 |
| ALT | C2 vs C3 | 3.845990 | 1.800999e-04 |
| SBP | C1 vs C2 | 4.162793 | 4.715674e-05 |
| SBP | C1 vs C3 | 9.020333 | 5.625544e-19 |
| SBP | C2 vs C3 | 3.938956 | 8.183701e-05 |
| DBP | C1 vs C2 | 3.703053 | 3.195311e-04 |
| DBP | C1 vs C3 | 6.610271 | 1.150853e-10 |
| DBP | C2 vs C3 | 2.155621 | 3.111330e-02 |
| RQ | C1 vs C2 | -2.942786 | 4.879100e-03 |
| RQ | C1 vs C3 | -3.075910 | 6.295843e-03 |
| RQ | C2 vs C3 | 0.363242 | 7.164242e-01 |
| REE | C1 vs C2 | 7.031894 | 3.056225e-12 |
| REE | C1 vs C3 | 13.961152 | 8.071443e-44 |
| REE | C2 vs C3 | 5.436721 | 5.427014e-08 |

Abbreviations: FFMI, fat-free mass index; BMI-SDS, body mass index standard deviation score; HOMA-IR, Homeostasis Model Assessment of Insulin Resistance; HOMA-β, Homeostasis Model Assessment of β-cell function; CRP, C-reactive protein; UR, uricemia; ALT, alanine aminotransferase; TGL, triglycerides; HDL. High Density Lipoprotein; SBP, systolic blood pressure; DBP, diastolic blood pressure; OGTT, oral glucose tolerance test; RQ, respiratory quotient; REE, resting expenditure energy.
