# Supplementary material for: Distinct metabolic phenotypes in adolescents with obesity identified by unsupervised learning: associations with insulin resistance and resting energy expenditure
Source: Front Endocrinol (Lausanne). 2026 Aug 27;17:1875870. doi: 10.3389/fendo.2026.1875870 (PMC13557994; doi:10.3389/fendo.2026.1875870)
Supplement: Supplementary file 4 [file Table4.docx]

**Supplementary Figure S1: Optimal number of clusters according to different methods**

**A. Optimal number of clusters (method = “silhouette”)**

**B. Optimal number of clusters (method = “elbow”)**

Number of cluster k

**C. Optimal number of clusters (method = “gap statistics”)**

Number of cluster k
